# Effectiveness of a Transdiagnostic Guided Internet-Delivered Protocol for Emotional Disorders Versus Treatment as Usual in Specialized Care: Randomized Controlled Trial

**DOI:** 10.2196/18220

**Published:** 2020-07-07

**Authors:** Alberto González-Robles, Amanda Díaz-García, Azucena García-Palacios, Pablo Roca, Josep Antoni Ramos-Quiroga, Cristina Botella

**Affiliations:** 1 Department of Basic and Clinical Psychology, and Psychobiology Universitat Jaume I Castellón de la Plana Spain; 2 CIBER Fisiopatología Obesidad y Nutrición (CIBERObn) Instituto Carlos III Madrid Spain; 3 Department of Personality, Assessment, and Clinical Psychology Universidad Complutense de Madrid Madrid Spain; 4 Department of Psychiatry Hospital Universitari Vall d'Hebron Barcelona Spain; 5 Group of Psychiatry, Mental Health and Addictions, Vall d'Hebron Research Institute (VHIR) Barcelona Spain; 6 Biomedical Network Research Centre on Mental Health (CIBERSAM) Barcelona Spain; 7 Department of Psychiatry and Forensic Medicine Universitat Autònoma de Barcelona Barcelona Spain

**Keywords:** transdiagnostic, internet, cognitive behavioral therapy, emotional disorders, depression, anxiety, specialized care

## Abstract

**Background:**

Anxiety disorders and depression (emotional disorders) are highly prevalent mental disorders. Extensive empirical evidence supports the efficacy of cognitive behavioral therapy (CBT) for the treatment of these disorders. However, there are still some barriers related to their dissemination and implementation, which make it difficult for patients to receive these treatments, especially in public health care settings where resources are limited. Recent advances in improving CBT dissemination encompass different perspectives. One is the transdiagnostic approach, which offers treatment protocols that can be used for a range of emotional disorders. Another approach is the use of the internet to reach a larger number of people who could benefit from CBT.

**Objective:**

This study aimed to analyze the effectiveness and acceptability of a transdiagnostic internet-delivered protocol (EmotionRegulation) with human and automated guidance in patients from public specialized mental health care settings.

**Methods:**

A 2-armed randomized controlled trial (RCT) was conducted to compare the effectiveness of EmotionRegulation with treatment as usual (TAU) in specialized mental health care. In all, 214 participants were randomly assigned to receive either EmotionRegulation (n=106) or TAU (n=108). Measurement assessments were conducted at pre- and postintervention and at a 3-month follow-up.

**Results:**

The results revealed the superiority of EmotionRegulation over TAU on measures of depression (*d*=0.41), anxiety (*d*=0.35), and health-related quality of life (*d*=−0.45) at posttreatment, and these gains were maintained at the 3-month follow-up. Furthermore, the results for expectations and opinions showed that EmotionRegulation was well accepted by participants.

**Conclusions:**

EmotionRegulation was more effective than TAU for the treatment of emotional disorders in the Spanish public mental health system. The implications of this RCT, limitations, and suggestions for future research are discussed.

**Trial Registration:**

ClinicalTrials.gov NCT02345668; https://clinicaltrials.gov/ct2/show/NCT02345668

## Introduction

### Disorder-Specific Cognitive Behavioral Therapy for Emotional Disorders

Anxiety and depressive disorders, also known as emotional disorders (EDs) [1], have the highest prevalence rates among psychological disorders [2,3], and are associated with substantial costs [4,5] and disability [6,7]. In the past three decades, research efforts to decrease the burden of these disorders have led to the development and evaluation of cognitive behavioral treatments in randomized controlled trials (RCTs) for each ED (ie, disorder-specific protocols), such as depression [8,9] and several anxiety disorders, including generalized anxiety disorder (GAD) [10,11], panic disorder (PD) and agoraphobia (AG) [12], social anxiety disorder (SAD) [13], and obsessive compulsive disorder (OCD) [14].

Although there is a large body of evidence showing the efficacy and effectiveness of *disorder-specific* cognitive behavioral therapy (CBT), in the past 15 years, an increasing number of researchers have agreed that there are some problems that hinder the optimal deployment of these treatments. The main drawback of disorder-specific treatments stems from the high comorbidity rates observed among anxiety disorders and between anxiety disorders and depressive disorders, with comorbidity estimates for these disorders ranging between 40% and 80% [15,16]. Thus, because disorder-specific treatment protocols focus on treating a specific diagnosis, the accompanying comorbid disorders do not receive therapeutic attention [17,18]. This problem becomes clearer when we take into account research linking comorbidity to aspects such as greater severity [3], increased chronicity rates [19], and a worse clinical course [20]. Another problem with disorder-specific treatments is that subthreshold symptoms that do not meet diagnostic thresholds for a particular disorder, but might be important to treat, normally go untreated [17]. Similarly, these protocols do not address diagnoses that do not fit any specific category, despite their clinical relevance, that is, *not otherwise specified* (NOS) anxiety and depressive disorders [15]. Finally, each disorder-specific treatment requires the use of different handbooks and protocols, which increases the economic costs and the amount of training needed to gain the knowledge and skills necessary to cover the array of anxiety and depressive disorders [17].

### Transdiagnostic Treatments for Anxiety and Depression

Transdiagnostic treatments have emerged as an alternative to the traditional disorder-specific approach that has dominated CBT research for the past 30 years. Transdiagnostic treatments have been developed and tested in several RCTs for anxiety disorders [21-24] and anxiety and depressive disorders [25-27], and their number continues to grow. Moreover, the efficacy and effectiveness of transdiagnostic treatments have been shown in different meta-analytic reviews, comparing them with different control groups, such as waiting list, attention control, and treatment as usual (TAU) [28-31], with pooled effect sizes (Hedges *g*) in the medium to large range for overall measures of anxiety (0.65-0.82) and depression (0.79-0.84). Moreover, an additional meta-analysis reported equivalent effects of transdiagnostic treatments (*g*=1.06) and disorder-specific treatments (*d*=0.95) on anxiety outcomes [32]. However, these meta-analyses include a number of mixed studies of different orientations, such as theory-based transdiagnostic treatments and tailored CBT. Therefore, these studies make it difficult to determine the effectiveness of each orientation (eg, the effectiveness of theory-based transdiagnostic treatments). The effectiveness of transdiagnostic treatments has also been shown in a meta-analysis by García-Escalera et al [33], with pooled effect sizes of *g*=0.80 for anxiety and *g*=0.72 for depression. Unlike the abovementioned meta-analyses, this study has the particularity that it only included theory-based transdiagnostic treatments. The main characteristic of theory-based transdiagnostic treatments, also known as *mechanistically transdiagnostic* treatments or transdiagnostic treatments based on *shared mechanisms* [34], is that they are designed to address the common psychopathological processes underlying anxiety and depression. Among the mechanistically transdiagnostic treatments for EDs, the Unified Protocol (UP) [35,36] stands out as one of the most empirically supported transdiagnostic protocols for anxiety and depression [23,37,38]. The UP is a CBT transdiagnostic protocol developed to address the underlying psychopathological processes that are common to anxiety and depressive disorders, with a particular focus on neuroticism, (low) extraversion, and emotion dysregulation, which have been shown to play a key role in the onset and maintenance of these disorders [39,40]. Thus, the main goal of the UP is to teach patients strategies to regulate their emotions in a more adaptive way through the following core treatment modules: (1) present-focused emotional awareness, (2) cognitive flexibility, (3) identification and prevention of emotional avoidance patterns, (4) increasing awareness and tolerance to emotion-elicited physical sensations, and (5) graded (interoceptive and situational) exposure procedures. The overall efficacy of the UP was first shown in an RCT where it was compared with a waitlist control group [23] and, more recently, in a larger RCT where it was compared with well-established disorder-specific CBT protocols for anxiety disorders [37]. In addition, some research shows the long-term effects of the UP [41] and its ability to produce changes in the temperament dimensions of behavioral inhibition (BI) and behavioral activation (BA) [42]. BI and BA have been conceptualized as 2 neurological systems representing motivational tendencies that are sensitive to threat and reward environmental cues, respectively [43]. These aspects have been intimately linked to neuroticism and negative affect and extraversion and positive affect [42]. Another treatment approach that includes principles or components that could be useful to target EDs is dialectical behavioral therapy (DBT) [44,45]. DBT initially emerged as a theoretical model and a treatment approach for the treatment of suicidal behaviors and borderline personality disorder, with the general aim of teaching strategies to change patterns of emotion dysregulation [46]. Of the range of strategies, DBT places a special emphasis on increasing experiential awareness and acceptance (mindfulness *what* and *how* techniques), but it also includes behavioral strategies such as the *opposite action*, designed to diminish distress by engaging in behaviors or actions opposite to those associated with negative emotions [44]. More recently, DBT skills have been adapted and successfully applied to several anxiety and depressive disorders [47,48], which suggests that they could be used in a transdiagnostic manner to improve the symptomatology of these disorders. For example, DBT principles may be combined with other evidence-based components (eg, components of the UP) to strengthen their effectiveness. The strategy of integrating CBT principles and components from different evidence-based therapies and orientations is consistent with the notion of process-based CBT, defined by Hofmann and Hayes [49]. As the authors state, “modern CBT places much less focus on protocols for syndromes and more focus on evidence-based processes linked to evidence-based procedures” [49].

### Internet-Delivered Interventions

In the past two decades, one of the most evident efforts made by researchers has been to take advantage of the possibilities offered by information and communication technologies to improve the assessment and treatment of psychological disorders. A clear example would be the use of the internet to increase the dissemination of empirically supported psychological treatments to anyone in need [50]. Research has shown promising and compelling evidence that internet-delivered psychological interventions are effective for a variety of psychosocial problems, including anxiety and depressive disorders [51,52]. The main advantages of internet-delivered treatments over traditional delivery methods (eg, face-to-face therapy) include widespread access and dissemination [53], a nonstigmatizing way of receiving psychological treatment [54], and increased cost-effectiveness [55].

Research has shown that transdiagnostic internet-delivered treatments are more effective than control groups [29] and that these treatments are at least as effective as individual and group face-to-face transdiagnostic treatments [33]. However, most of the existing literature on transdiagnostic treatments is limited to studies conducted in community settings, with few studies carried out in public contexts such as primary or specialized care [21]. Indeed, to our knowledge, no transdiagnostic internet-delivered treatments for anxiety and depression have been conducted in specialized public mental health care. This is somewhat surprising because transdiagnostic internet-delivered treatments in this particular setting could have several advantages for both clinicians and the patients attending these centers. First, anxiety and depressive disorders are disorders with the highest prevalence rates [2,3]. Second, resources in these settings are usually scarce, which affects both the quantity and the quality of the mental health care provided [56]. Third, a large percentage of patients with anxiety and depressive disorders do not receive treatment in mental health care centers [57]. In the specific case of Spain, most patients attending public mental health units suffer from anxiety and depressive disorders [58], the ratio of clinical psychologists to patients is one of the lowest in Europe [59], and patients have to endure long waitlists to receive treatment [60]. Fourth, transdiagnostic treatments can be provided at a lower cost (eg, in terms of training) [61]. Finally, the use of the internet can help improve mental health services, for instance, by reducing the waiting period to receive face-to-face treatment [62] or by implementing these treatments as part of a stepped-care model that takes patients’ profiles and needs into account. Thus, each patient can be assigned the most appropriate treatment [63], leaving face-to-face therapy for those patients who are less likely to benefit from internet-delivered interventions.

### This Study

Taking all of this into consideration, in this study, an RCT was conducted to test the effectiveness of a transdiagnostic internet-delivered protocol for ED (EmotionRegulation), compared with TAU provided in Spanish public specialized mental health care. EmotionRegulation includes components of the UP and the skills from DBT (eg, mindfulness *what* and *how* techniques), and it was designed to target a wide range of EDs, including major depressive disorder (MDD), dysthymic disorder (DD), PD, AG, SAD, GAD, OCD, anxiety NOS, and depression NOS. It was hypothesized that (1) the EmotionRegulation group would outperform the TAU group on measures of overall anxiety and depression, temperament (ie, BI and BA), and health-related quality of life (QoL) at posttreatment; (2) these posttreatment changes would be maintained at follow-up; (3) a significantly greater clinical change would be observed in EmotionRegulation compared with TAU; and (4) participants in the EmotionRegulation group would rate the treatment as acceptable (scores on expectations and opinion ≥7/10).

## Methods

### Study Design

A 2-armed RCT was conducted. Participants were randomly assigned in a 1:1 ratio to one of the following 2 conditions: (1) EmotionRegulation and (2) TAU. Participants were stratified by principal diagnosis, performing block randomization in blocks of 4 to ensure that all the principal diagnoses were equally represented across conditions. Computer-generated random number sequences were obtained using statistical software (Epidat 4.1, SourceForge). This task was performed by an independent researcher who was unaware of the characteristics of the study.

The study was conducted in compliance with the study protocol, the Consolidated Standards of Reporting Trials (CONSORT) statement [64,65], the CONSORT of Electronic and Mobile HEalth Applications and onLine TeleHealth guidelines [66], and the Declaration of Helsinki and good clinical practice. A full description of the study protocol has been reported elsewhere [67]. The RCT obtained ethical approval from the Ethics Committee of Universitat Jaume I (Castellón, Spain) and the Clinical Research Ethics Committees of 3 hospitals (Consorcio Hospitalario Provincial de Castellón, Hospital Universitario de la Ribera, and Hospital Universitario Vall d’Hebron). The study protocol was registered at ClinicalTrials.gov (NCT02345668) on July 27, 2015. The duration of the intervention period was 18 weeks for participants in both conditions, and participants’ assessments were conducted at pre- and posttreatment and at 3- and 12-month follow-ups. Both the intervention (EmotionRegulation) and the assessment instruments, except the diagnostic interview, were delivered through a web platform designed by our research group [68]. All transferred data were secured via Advanced Encryption Standard-256 encryption. Study researchers conducting posttreatment and follow-up assessments (ie, diagnostic interviews) were blinded to the participants’ treatment conditions. To ensure blinding of the evaluators, participants were informed that an independent researcher would contact them to conduct follow-up assessments, and they were asked not to disclose the treatment condition to which they had been allocated. This study reports pre- to posttreatment data and data at 3-month follow-up.

### Sample Size

Several studies were considered for the calculation of the expected sample size [23,69,70]. On the basis of a minimum power of 0.80 in a 1-tailed test (ie, *t* test for differences between 2 independent means), an α of .05, and an estimated dropout rate of approximately 30%, a sample size of 78 participants per condition was determined to detect a posttreatment effect size of 0.40 (Cohen *d*) between the 2 conditions. In addition, based on the literature [71,72], an estimated dropout rate of approximately 30% was expected. Thus, the final sample size was set at 100 participants per condition (total of 200 participants). The G*Power software (version 3.1.9.4, Heinrich-Heine-Universität) was used to calculate the sample size [73].

### Participants

Participants were recruited from adult outpatients attending Spanish public specialized mental health care services (mental health units) to seek psychological and/or psychiatric treatment between July 2015 and June 2019. Initial recruitment was performed by clinical psychologists and psychiatrists working in these centers, and it took place in 3 different hospitals: Consorcio Hospitalario Provincial de Castellón (Castellón de la Plana), Hospital Universitario de la Ribera (Valencia), and Hospital Universitario Vall d’Hebrón (Barcelona). Recruitment was performed as follows: (1) once psychiatrists and clinical psychologists had identified a potential candidate, they offered the patient the possibility of participating in the study and described the study characteristics to him or her; (2) patients who were interested in participating gave their informed written consent, and the clinician filled out a document with the participants’ sociodemographic and clinical characteristics (moreover, in this stage, participants were provided with a document containing information about the study); (3) one of the researchers involved in the study contacted the participants by phone to schedule a face-to-face appointment to evaluate eligibility criteria using a structured diagnostic interview; and (4) whenever a participant met the eligibility criteria, an independent researcher (unaware of the study characteristics) was contacted to implement randomization, and participants completed the remaining assessment instruments (self-reported questionnaires) through web-based surveys.

Participants were selected based on the following inclusion criteria: (1) aged 18 years or older; (2) ability to understand and read Spanish; (3) having access to the internet at home and an email address; (4) meeting Diagnostic and Statistical Manual of Mental Disorders, Fourth Edition (DSM-IV) diagnostic criteria [74] for ED (ie, MDD, DD, depression NOS, PD, AG, SAD, GAD, anxiety NOS, and OCD); (5) providing written informed consent; (6) not suffering from a severe mental disorder (schizophrenia, bipolar disorder, and alcohol and/or substance dependence disorder); (7) not presenting a high risk of suicide; (8) not suffering from a disabling medical disease that prevented the participant from carrying out the psychological treatment; and (9) not receiving another psychological treatment during the study (in the experimental group). Pharmacological treatment was allowed, but participants had to be taking the same dose during the 2 months before enrolling in the study. In addition, participants in the experimental group whose medication was increased or changed during the study period were excluded from the trial (decreases in pharmacological treatment were accepted). There was no monetary compensation for participation in the study under any of the treatment conditions.

### Instruments

#### Clinical Outcomes

##### Diagnosis Interview

Clinical diagnoses were obtained using the mini-international neuropsychiatric interview (MINI) version 5.00 [75,76], a brief structured diagnostic psychiatric interview for the assessment of key DSM-IV and International Classification of Diseases, 10th Revision, diagnoses.

##### Principal Outcomes

###### Beck Depression Inventory, Second Edition

Beck depression inventory, second edition (BDI-II) [77,78], is a self-report questionnaire with 21 items about the different symptoms characterizing MDD, added together to obtain the total score, which can be a maximum of 63 points. The instrument has shown good internal consistency (α=.76-.95). The Spanish version also showed high internal consistency (α=.87) for both the general and clinical populations (α=.89). Cronbach α for the BDI-II in this study was .90.

###### Beck Anxiety Inventory

The Beck anxiety inventory (BAI) [79,80] is a 21-item self-report scale that assesses anxiety, with a maximum score of 63 points. Each item has a 4-point severity scale (from *not at all* to *severely*) that addresses symptoms experienced during the previous week. Previous validation studies have shown an internal consistency ranging from 0.85 to 0.94 as well as convergent and divergent validity. The Spanish version of the BAI has demonstrated high internal consistency (α=.93). Cronbach α for the BAI in this study was .92.

##### Secondary Outcomes

###### Behavioral Inhibition Scale and Behavioral Activation Scale

The behavioral inhibition scale (BIS) and behavioral activation scale (BAS) [81,82] contains 20 items rated from 1 to 4, with 7 BIS subscale items that evaluate emotional responses of individuals to impending negative events and 13 BAS items that evaluate the behavioral and emotional responses of individuals to potentially positive events. The BIS and BAS have shown good reliability in individuals with EDs (α=.73-.92) and good convergent and discriminant validity as indicators of temperament. The internal consistency of the Spanish version ranges between 0.65 and 0.82. Cronbach α for the BIS and BAS subscales in this study were .61 and .80, respectively.

###### Quality of Life EuroQoL-5D-3L Questionnaire

Quality of life EuroQoL-5D-3L (EQ-5D-3L) questionnaire [83,84] is a generic instrument that measures health-related QoL and consists of 2 parts. Part 1 assesses self-reported problems in each of the following 5 domains: mobility, self-care, daily activities, pain/discomfort, and anxiety/depression. Each domain is divided into 3 levels of severity corresponding to no problems, some problems, and extreme problems, yielding a population-based preference score or societal index (SI). A total of 243 theoretically possible health states can be obtained, and the SI is calculated on the basis of these health states. Values range from 1 (best health state) to 0 (death). However, this index may also provide negative values that correspond to health states perceived as worse than death. Utility scores for these health states were assigned using the available Spanish population tariffs. Part 2 records the subjects’ self-assessed health on a visual analog scale (VAS), a 10-cm vertical line on which the best and worst imaginable health states score 100 and 0, respectively. In this study, health-related QoL was assessed using the VAS.

###### Disorder-Specific Measures

Disorder-specific symptoms were evaluated using 4 different self-report questionnaires. Symptoms of GAD were assessed using the Penn State Worry Questionnaire [85,86]. PD and AG symptoms were evaluated using the Panic Disorder Severity Scale, Self-Reported [87,88]. SAD symptoms were evaluated using the Social Interaction Anxiety Scale [89,90], and OCD symptoms were assessed using the Obsessive Compulsive Inventory-Revised [91,92]. All 4 instruments have shown adequate psychometric properties in both the original and Spanish validations. Cronbach αs for these scales in this study were .78, .89, .80, and .91, respectively. More details about these assessment instruments have been described elsewhere [67].

#### Treatment Acceptability

##### Expectations and Opinions of Treatment Scales

These questionnaires were adapted from the study by Borkovec and Nau [93]. Each scale is made up of 5 items, rated from 0 (*nothing at all*) to 10 (*completely*), which ask how logical the treatment seems to be (*How logical do you* *think this treatment is?*), to what extent it satisfies the patient (*How satisfied are you with the treatment?*), whether the patient would recommend it to a person with the same problem (*To what extent do you feel confident recommending this treatment to a friend who has the same problems?*), whether it could be used to treat other psychological problems (*To what extent do you think this treatment could be useful in treating other psychological problems?*), and its usefulness for the patient’s problem (*To what extent do you think this treatment will be/was helpful to you?*). The expectation scale was applied after the treatment rationale was explained. Its objective is to measure subjective patient expectations regarding this treatment. The opinion scale was administered when the patient had completed the treatment, and it was designed to assess satisfaction with this treatment.

### Treatments

#### EmotionRegulation

Following randomization, participants in the treatment condition were contacted via telephone by a researcher who provided free access to EmotionRegulation, a 12-module transdiagnostic internet-delivered protocol for the treatment of ED, namely, MDD, DD, depression NOS, PD, AG, GAD, SAD, anxiety NOS, and OCD. The protocol is delivered through a web platform [68] designed by our research group. Access to the web platform is through a unique username-password combination and is available 24 hours a day. The treatment was first developed as a manualized transdiagnostic treatment protocol with handbooks for both patient and therapist and then adapted to be delivered through a web-based platform. The web platform has successfully been used in previous RCTs exploring the efficacy of internet-delivered treatments for several disorders, such as depression [94] and flying phobia [95].

The main core components are based on the UP [35,36], but the protocol also contains treatment strategies derived from DBT [46]. The principal aim of the treatment components in EmotionRegulation is to learn and practice adaptive ways to regulate emotions from a transdiagnostic perspective, with the following treatment components: present-focused emotional awareness, cognitive flexibility, emotional avoidance and emotion-driven behaviors, and exposure procedures (interoceptive and situational). The protocol also contains a module to facilitate the patient’s engagement with the therapy (motivation for change), a module with psychoeducation about emotions, and a relapse prevention module. Regarding the DBT components, greater emphasis was placed on the present-focused emotional awareness component by adapting and including strategies such as the *what* and *how* techniques and the concept of radical acceptance. Moreover, the opposite action was integrated into the treatment as a way to address emotion-driven behaviors. EmotionRegulation includes a Welcome module that contains general information about the protocol and its goals as well as recommendations for benefiting from it and 12 treatment modules (described in Table 1).

The modules are sequential to enable step-by-step movement through the program. The program duration can vary among the users, and participants had access to the protocol for a maximum period of 18 weeks. Moreover, participants were allowed to use the program any time they wanted during the trial period (ie, during the follow-up periods).

Regarding guidance, all participants in this condition received therapist and automated support. Therapist support consisted of (1) an initial face-to-face session to explain the characteristics of the study and administer the diagnostic interview to confirm the eligibility criteria, (2) an initial phone call encouraging participants to start the intervention after the baseline assessments had been completed, (3) 1 weekly brief phone call (maximum of 10 min) during the treatment period, and (4) a final phone call (once the treatment had ended) to remind participants that they would be allowed to use the program at any time during the trial period and that they would be contacted for follow-up assessments. Automated support consisted of 2 weekly text messages reminding participants about the importance of completing the homework tasks and encouraging them to review the treatment modules. Text messages were sent through a secure web platform [96]. This web platform was only used to send text messages (unidirectionally) with predefined contents, and they did not include personal information that could have identified the participants. Additional details about the treatment and support protocols, as well as other functionalities of the web-based platform, have been published elsewhere [67]. Finally, it is important to note that all participants in this condition were allowed to continue to receive TAU, but only for monitoring of pharmacological treatment. Participants who received other forms of treatment during the study period (eg, psychological treatment) or who experienced increases or changes in pharmacological treatment were excluded from the analyses.

**Table 1:** Treatment modules and their objectives.

Module	Objective
1. Introduction to treatment	Provides a framework about the role of emotion regulation in ED^a^.
2. Motivation for change and goal setting	To analyze pros and cons of changing, emphasize the importance of being motivated, and help to establish significant life goals.
3. Understanding the role of emotions	Provides psychoeducation about the roles and functions of emotions and trains the patient to track the 3 components of emotional experiences.
4. Nonjudgmental emotional awareness and acceptance of emotional experiences	Aims to train the patient in nonjudgmental emotional awareness (ie, mindfulness *what* and *how* skills) and the acceptance of emotional experiences.
5. Practicing present-focused awareness	To continue to practice the acceptance of emotional experiences and increase awareness of physical sensations, thoughts, emotions, and daily activities.
6. Learning to be flexible	Focuses on the identification of maladaptive ways of thinking (ie, thinking traps).
7. Practicing cognitive flexibility	Aims to teach the patients strategies to modify thinking traps (ie, cognitive reappraisal). It also provides information about intrusive thoughts and how to deal with them.
8. Emotional avoidance	Aims to teach the patients to identify the emotion avoidance strategies that contribute to the maintenance of ED.
9. Emotion-driven behaviors	To learn the concept of EDBs^b^ and replace their maladaptive EDB with other more adaptive behaviors.
10. Accepting and facing physical sensations	To teach the role of physical sensations in the emotional response and provide training in interoceptive exposure.
11. Facing emotions in the contexts in which they occur	To build exposure hierarchies to help the patients begin to face situation-elicited avoided emotions.
12. Relapse prevention	To review what patients have learned throughout the program, schedule the future practice of the learned strategies, and teach the patient how to identify and cope with future high-risk situations.

^a^ED: emotional disorder.

^b^EDBs: emotion-driven behaviors.

#### Treatment as Usual

TAU was treatment as delivered in current daily practice by psychiatrists and clinical psychologists in the mental health centers in Spain. TAU in this study was provided by 3 hospitals: Consorcio Hospitalario Provincial de Castellón (Castellón de la Plana), Hospital Universitario de la Ribera (Valencia), and Hospital Universitario Vall d’Hebron (Barcelona). To maximize the external validity of this RCT, participants in this condition were allowed to receive either psychiatric treatment (ie, prescription and monitoring of antidepressant and/or anxiolytic medication), psychological treatment (including case management, group psychotherapy, empathic listening, and/or supportive counseling), or a combination of both. The frequency of visits during the 18-week treatment period varied depending on the type of treatment (ie, psychiatric or psychological) provided to the participant. Patients in the TAU condition who were already receiving any of the aforementioned treatments at the time of enrollment were informed that they would continue to receive these services during the treatment period. Furthermore, participants receiving a treatment other than those provided in the mental health unit were excluded from the trial. All participants allocated to TAU were offered free access to the treatment platform after the study ended.

### Therapists and Treatment Fidelity

The treatment and support protocols were administered by doctoral students with at least two years of experience in the diagnosis, psychological assessment, and application of CBT for different ED. Several steps were taken to ensure treatment fidelity. First, therapists had previously been trained in the application of the treatment modules. Second, a support protocol (ie, weekly phone calls and automated text messages) was developed to be applied to all the participants in the EmotionRegulation condition. This support protocol has been briefly described earlier, but more details can be found in the study by González-Robles et al [67]. Third, to increase diagnosis reliability, all therapists involved in the participants’ assessment were trained in the application of the diagnostic interview (MINI).

### Data Analysis Plan

All analyses were performed using the Statistical Package for Social Sciences version 25.

First, chi-square tests for categorical data and independent samples *t* tests for continuous data were performed to confirm that there were no significant differences between the groups at baseline on any of the sociodemographic and clinical variables.

Intention-to-treat (ITT) analyses were performed following Newman’s guidelines [97], using maximum likelihood (ML) estimation through the expectation maximization imputation method. To handle missing data, we followed the procedure suggested by Hair et al [98]. First, we explored the types of missing data and determined that data were missing at the construct level. On the basis of this, we concluded that the data were susceptible to imputation. Second, the quantity of missing data was analyzed to ensure that none of the measures exceeded the recommended limits to implement this method [99]. Third, Little’s missing completely at random (MCAR) tests were carried out to analyze the pattern of missing data, concluding that all missing data were MCAR (X^2^_25_=28.7; *P*=.28). Finally, a sensitivity analysis was performed on the main outcomes to compare the results of the per-protocol sample (ie, completers) with the imputed values. This analysis revealed that the ML estimation was not likely to produce biased estimations in the main analyses, reaching the same conclusions in both the completers and the imputed dataset (per-protocol: *F*_BAI (1,127)_=6.1; *P*=.02; and *F*_BDI (1,127)_=15.54; *P*<.001; ITT: *F*_BAI (1,197)_=4.79; *P*=.03; and *F*_BDI (1,197)_=12.97; *P*<.001).

To test the first hypothesis and control for baseline differences, analyses of covariance (ANCOVAs) were performed to compare the effects of the groups on measures of anxiety, depression, temperament, and health-related QoL, taking condition as the between-subject variable and the pretreatment scores as covariates. The use of ANCOVAs for the analysis has been recommended by several authors as a more powerful tool to analyze data in studies with randomized designs [100,101].

To test the second hypothesis, a 2 (condition: EmotionRegulation vs TAU) × 3 (time: pretreatment vs posttreatment vs 3-month follow-up) mixed analysis of variance (ANOVA) was performed to test whether the differences between EmotionRegulation and TAU (ie, between-subjects factors) were maintained at follow-up (ie, within-subject factor). The following assumptions for the mixed ANOVA were analyzed: normality (Shapiro-Wilk test), homoscedasticity (Levene test), independence (nonparametric Runs test), and sphericity (Mauchly test). The degrees of freedom were corrected using Greenhouse-Geisser whenever the sphericity assumption was violated. Moreover, pairwise Bonferroni-corrected tests were used for posthoc comparisons.

To compute the magnitude of both within-group and between-group changes, effect sizes (Cohen *d*) were calculated by dividing the differences between means by the pooled SD. Effect sizes were interpreted according to Cohen convention: effect sizes of 0.20 are considered low, effect sizes of 0.50 are considered medium, and effect sizes of 0.80 and above are considered large [102].

To test the third hypothesis, we explored the clinical significance of the changes achieved by the participants as well as potential deterioration rates using Jacobson and Truax’s reliable change index (RCI) [103] for the main outcome measures (BDI-II and BAI) in the completer sample for posttreatment and follow-up measurements. First, the cutoff points for the posttreatment and follow-up scores were determined to be within the range of a functional distribution. The RCI was then calculated to test the clinically significant change, with an RCI of |1.96| or greater (*P*<.05). Finally, both criteria were taken into account to classify participants into the following 4 categories: (1) recovered: when the change is significantly reliable (RCI≥|1.96|; *P*<.05) and the posttreatment score is located within the range of the functional distribution (mean [SD 2]), (2) improved: when the change is significantly reliable but the posttreatment score is below the functional level, (3) not changed: when the change is not significantly reliable and the posttreatment score does not reach the functional level, and (4) deteriorated: when the change is significantly reliable but the posttreatment score is worse than the pretreatment score.

Finally, to test the fourth hypothesis, the scores on expectations and opinions were analyzed by calculating means and SDs for each of the items on the expectation and opinion of treatment scales. In addition, 1-way ANOVAs were performed to analyze the significance of the differences between expectations and opinions.

## Results

### Participant Flow and Attrition

**Figure 1:**
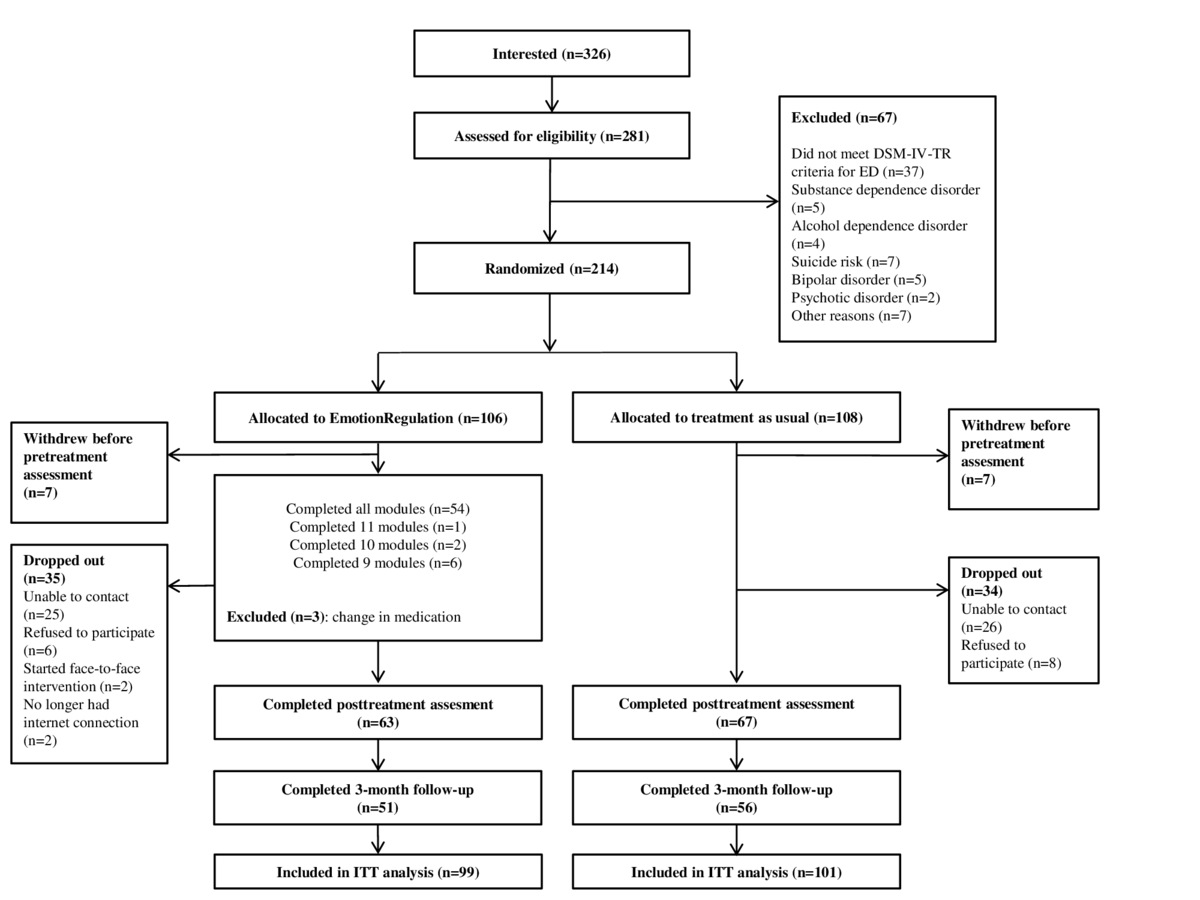
Flowchart of participants. ED: emotional disorder; DSM-IV-TR; Diagnostic and Statistical Manual of Mental Disorders, Fourth Edition, Text Revision; ITT: intention-to-treat.

A flowchart of the study participants is displayed in Figure 1. A total of 326 patients expressed interest in the study, 281 of whom were assessed for eligibility. Of these 281, 67 participants were excluded from the study. A total of 214 participants were randomized to either EmotionRegulation (n=106) or TAU (n=108). In addition, 7 patients in each condition withdrew from the study before the pretreatment assessment. Consequently, these participants were not included in any of the analyses.

Regarding attrition, 35 participants in the EmotionRegulation condition (35/106, 33.0%) and 34 in the TAU condition (34/108, 31.5%) dropped out of the study (reasons for dropout are shown in Figure 1). In addition, 3 participants in the EmotionRegulation condition had to be excluded from the trial because of a change in their pharmacological treatment during the treatment period. Posttreatment data were obtained from 63 participants (63/99, 64%) in the EmotionRegulation condition and from 67 participants (67/101, 66.3%) in the TAU condition. Follow-up data were collected from 51 participants (51/99, 52%) in the EmotionRegulation condition and 56 participants (56/101, 55.4%) in the TAU condition. Finally, 99 participants in the EmotionRegulation group and 101 participants in the TAU condition were included in the ITT analysis.

### Baseline Characteristics

Participants (N=200) had a mean age of 38.44 years (SD 10.80; range 18-68), and they were mostly females (138/200, 69.0%). Table 2 provides the sociodemographic and clinical characteristics for both conditions at baseline. There were no significant differences between the EmotionRegulation and TAU groups at baseline on any of the sociodemographic and clinical characteristics. Moreover, no significant differences were found for medication, principal diagnosis, number of comorbid diagnoses, or clinical severity on any of the measures.

**Table 2:** Demographic and clinical characteristics of the sample at baseline (N=200).

Variable		EmotionRegulation regulation (n=99)	Treatment as usual (n=101)	X^2^ (*df*)	*t* test (*df*)	*P* value
Age (years), mean (SD)		38.64 (10.61)	38.25 (11.03)	N/A^a^	0.25 (198)	.80
**Sex, n (%)**				**1.3 (1)**	**N/A**	**.26**
	Female	72 (72)	66 (65.3)			
	Male	27 (27)	35 (34.7)			
**Marital status, n (%)**				**1.1 (3)**	**N/A**	**.78**
	Single	22 (22)	26 (25.7)			
	Married or partnered	63 (63)	65 (64.4)			
	Divorced or widowed	14 (14)	10 (9.9)			
**Education, n (%)**				**2.1 (2)**	**N/A**	**.35**
	Basic studies	26 (26)	36 (35.6)			
	Secondary studies	41 (41)	35 (34.7)			
	University studies	32 (32)	30 (29.7)			
**Occupation, n (%)**				**3.3 (6)**	**N/A**	**.77**
	Student	9 (9)	11 (10.9)			
	Housekeeper	6 (6)	9 (8.9)			
	Employed	45 (45)	36 (35.6)			
	Unemployed	23 (23)	22 (21.8)			
	Off work	13 (13)	17 (16.8)			
	Retired	3 (3)	6 (5.9)			
**Monthly income (€), n (%)**				**1.0 (4)**	**N/A**	**.91**
	None	27 (27)	28 (27.7)			
	<641.40 (US $699.45)	20 (20)	16 (15.8)			
	641.40-1282.80 (US $699.46-1398.89)	32 (32)	38 (37.6)			
	1282.81-2565.60 (US $1398.90-2797.78)	18 (18)	17 (16.8)			
	>2565.60 (US $2798.78)	2 (2)	2 (2.0)			
**Principal diagnosis, n (%)**				**2.7 (8)**	**N/A**	**.95**
	GAD^b^	23 (23)	26 (27.7)			
	AG^c^	16 (16)	13 (12.9)			
	PD^d^	9 (9)	5 (5.0)			
	SAD^e^	4 (4)	4 (4.0)			
	OCD^f^	8 (8)	12 (12.0)			
	MDD^g^	20 (20)	22 (21.8)			
	DD^h^	7 (7)	6 (5.9)			
	Anxiety NOS^i^	10 (10)	9 (8.9)			
	Depression NOS	2 (2)	3 (3.0)			
**Comorbid diagnoses, n**				**N/A**	**N/A**	**N/A**
	GAD	10	18			
	PD	6	5			
	AG	18	22			
	SAD	7	10			
	OCD	2	4			
	MDD	15	18			
	DD	15	5			
	Anxiety NOS	3	1			
	Depression NOS	1	0			
	Alcohol abuse	1	2			
	Substance abuse	0	2			
**Number of comorbid disorders, n (%)**				**2.3 (3)**	**N/A**	**.50**
	0	49 (49)	41 (40.6)			
	1	29 (29)	38 (37.6)			
	2	15 (15)	13 (12.9)			
	≥ 3	6 (6)	8 (7.9)			
**Medication, n (%)**				**5.2 (3)**	**N/A**	**.16**
	None	29 (29)	18 (17.8)			
	Antidepressant	22 (22)	20 (19.8)			
	Anxiolytic	10 (10)	17 (16.8)			
	Both	38 (38)	46 (45.5)			

^a^N/A: not applicable.

^b^GAD: generalized anxiety disorder.

^c^AG: agoraphobia.

^d^PD: panic disorder.

^e^SAD: social anxiety disorder.

^f^OCD: obsessive compulsive disorder.

^g^MDD: major depressive disorder.

^h^DD: dysthymic disorder.

^i^NOS: not otherwise specified.

### Amount of Support Provided

Participants in the EmotionRegulation condition were provided a mean of 49.97 min (SD 41.20 min) of clinician support delivered through phone calls. In addition, an initial face-to-face session was scheduled with all the patients in both conditions (EmotionRegulation and TAU) to explain the study and perform the screening assessment, with an approximate duration of 60 min for each participant. Regarding automated support, that is, weekly text messages, participants in the EmotionRegulation group were sent a mean of 24.61 text messages (SD 8.80).

### Effectiveness of EmotionRegulation on Primary and Secondary Outcome Measures

Table 3 provides the means and SDs for the 2 conditions at pre- and posttreatment and at 3-month follow-up on both primary and secondary outcome measures. Additional data on change scores and within-group effect sizes for diagnosis-specific measures are reported in Multimedia Appendix 1.

**Table 3:** Descriptive statistics for EmotionRegulation and treatment as usual at pretreatment, posttreatment, and 3-month follow-up.

Instrument	EmotionRegulation (n=99), mean (SD)			Treatment as usual (n=101), mean (SD)		
	Pre-T^a^	Post-T^b^	F/U^c^	Pre-T	Post-T	F/U
Beck depression inventory-II	23.49 (11.01)	15.54 (10.9)	15.70 (11.97)	24.08 (11.69)	19.85 (12.85)	17.90 (13.23)
Beck anxiety inventory	20.00 (11.88)	15.08 (10.12)	15.41 (10.50)	22.27 (12.93)	18.88 (11.31)	18.11 (11.21)
Behavioral inhibition and behavioral activation scale—behavioral inhibition system	23.32 (2.76)	22.30 (2.67)	21.81 (2.67)	23.40 (2.87)	22.87 (2.44)	22.44 (2.42)
Behavioral inhibition scale and behavioral activation scale—behavioral activation system	35.26 (5.93)	36.26 (5.31)	34.94 (5.27)	35.84 (5.58)	35.01 (5.98)	34.07 (6.04)
EuroQoL-5D-3L questionnaire	55.86 (16.72)	65.38 (14.63)	63.12 (15.18)	53.56 (18.25)	58.02 (17.46)	57.81 (17.28)

^a^Pre-T: pretreatment.

^b^Post-T: posttreatment.

^c^F/U: follow-up.

#### Pretreatment to Posttreatment Effects

##### Principal Outcome Measures

The ANCOVAs of the baseline-corrected postintervention scores revealed a significant condition effect on anxiety (BAI: *F*_1,197_=4.79; *P*=.03; *η^2^* partial=0.02) and depression (BDI-II: *F*_1,197_=12.97; *P*<.001; *η^2^* partial=0.06), reflecting that the EmotionRegulation group showed significantly lower posttreatment anxiety and depression scores than the TAU group.

##### Secondary Outcome Measures

Regarding the measures of BI and BA (BISBAS), the ANCOVAs yielded a significant condition effect for the BA dimension (BAS: *F*_1,197_=9.66; *P*=.002; *η^2^* partial=0.05), indicating that patients in the EmotionRegulation group had significantly higher BA scores than those in the TAU group. Although patients in the EmotionRegulation group showed greater improvements in the BI dimension, no significant differences between groups were observed for this dimension (BIS: *F*_1,197_=2.44; *P*=.12; *η^2^* partial=0.01). On the other hand, the ANCOVA revealed a significant condition effect on health-related QoL (EQ-5D-3L: *F*_1,197_=10.38; *P*=.001; *η^2^* partial=0.05), indicating that health-related QoL scores were significantly higher in the EmotionRegulation group posttreatment than in the TAU group.

#### Follow-Up Effects

##### Principal Outcome Measures

For depression, a significant condition×time interaction effect was found (*F*_2,396_=6.18; *P*=.01; *η^2^* partial=0.03). There was a significant time effect (*F*_2,396_=102.07; *P*<.001; *η^2^* partial=0.34) and a nonsignificant condition effect on anxiety scores (*F*_1,198_=2.25; *P*=.14; *η^2^* partial=0.01). In the EmotionRegulation condition, Bonferroni tests indicated that the differences between pre- and posttreatment were significant (*P*<.001), but the differences between posttreatment and follow-up were not significant (*P*>.99), revealing that the reductions in depression scores were maintained at the 3-month follow-up.

The analyses showed no condition×time interaction effect on anxiety (*F*_1.62,319.88_=0.75; *P*=.45; *η^2^* partial=0.004). However, there was a significant time effect (*F*_1.62,319.88_=29.35; *P*<.001; *η^2^* partial=0.13) and a significant condition effect on anxiety scores (*F*_1,198_=4.20; *P*=.04; *η^2^* partial=0.02). Although there was no interaction effect, we decided to perform posthoc tests to preliminarily explore the direction of the changes. In the EmotionRegulation group, posthoc comparison tests revealed significant differences between pre- and posttreatment (*P*<.001), but no significant differences between posttreatment and follow-up in the EmotionRegulation group (*P*>.99).

##### Secondary Outcome Measures

Regarding temperament measures, no significant condition×time interaction effect was found for the BIS subscale (*F*_1.49,295.29_=0.93; *P*=.34; *η^2^* partial=0.005). A significant time effect was found (*F*_1.49,295.29_=15.33; *P*<.001; *η^2^* partial=0.07), but the effect of condition was not significant (*F*_1,198_=2.51; *P*=.12; *η^2^* partial=0.01).

For the BAS subscale, a significant condition×effect interaction effect was found (*F*_1.84,363.40_=5.62; *P*=.02; *η^2^* partial=0.03). In the EmotionRegulation group, posthoc tests showed that BAS scores were significantly higher at posttreatment than at baseline (*P*=.05). However, these differences vanished at follow-up, as shown by the comparison between pretreatment and follow-up scores (*P*>.99).

Regarding health-related QoL, the analyses did not reveal a condition×time interaction effect (*F*_1.7,336.93_=2.73; *P*=.08; *η^2^*partial=0.01). However, there was a significant time effect (*F*_2,396_=23.34; *P*=.001; *η^2^* partial=0.11) and a significant condition effect (*F*_1,198_=6.3; *P*=.01; *η^2^* partial=0.03), which indicated a significant improvement in health-related QoL at posttreatment in both EmotionRegulation and TAU and generally higher health-related QoL in the EmotionRegulation group. Table 4 provides the effect sizes for the within- and between-group comparisons.

**Table 4:** Within- and between-group effect sizes and 95% CIs.

Instrument	EmotionRegulation (n=99), *d* (95% CI)		TAU^a^ (n=101), *d* (95% CI)			EmotionRegulation versus TAU, *d* (95% CI)	
	Pre-post	Pre-F/U^b^	Pre-post	Pre-F/U	Posttreatment		F/U
Beck depression inventory-II	0.72^c^ (0.54 to 0.90)	0.70 (0.54 to 0.87)	0.36 (0.23 to 0.49)	0.52 (0.39 to 0.66)	0.41 (0.13 to 0.69)		0.24 (−0.04 to 0.52)
Beck anxiety inventory	0.41 (0.25 to 0.57)	0.38 (0.20 to 0.56)	0.26 (0.09 to 0.43)	0.32 (0.13 to 0.51)	0.35 (0.07 to 0.63)		0.25 (−0.03 to 0.53)
Behavioral inhibition scale and behavioral activation scale—behavioral inhibition system	0.37 (0.10 to 0.63)	0.54 (0.26 to 0.82)	0.18 (−0.07 to 0.44)	0.33 (0.06 to 0.61)	0.22 (−0.06 to 0.50)		0.25 (−0.03 to 0.52)
Behavioral inhibition scale and behavioral activation scale—behavioral activation system	−0.17^c^ (−0.31 to −0.03)	0.05 (−0.09 to 0.20)	0.15 (0.01 to 0.29)	0.31 (0.15 to 0.48)	−0.22 (−0.50 to 0.06)		−0.15 (−0.43 to 0.12)
EuroQoL-5D-3L questionnaire	−0.57 (−0.76 to −0.37)	−0.43 (−0.64 to −0.22)	−0.24 (−0.44 to −0.04)	−0.23 (−0.44 to −0.02)	−0.45 (−0.74 to −0.17)		−0.33 (−0.60 to −0.05)

^a^TAU: treatment as usual.

^b^F/U: 3-month follow-up.

^c^Positive effect sizes denote a decrease in scores, whereas negative effect sizes denote an increase.

### Significance of Clinical Improvements

The results for the significance of the clinical changes on measures of overall depression and anxiety (BDI-II and BAI, respectively) are summarized below.

#### Changes in Depression

##### Baseline to Posttreatment Changes

**Figure 2:**
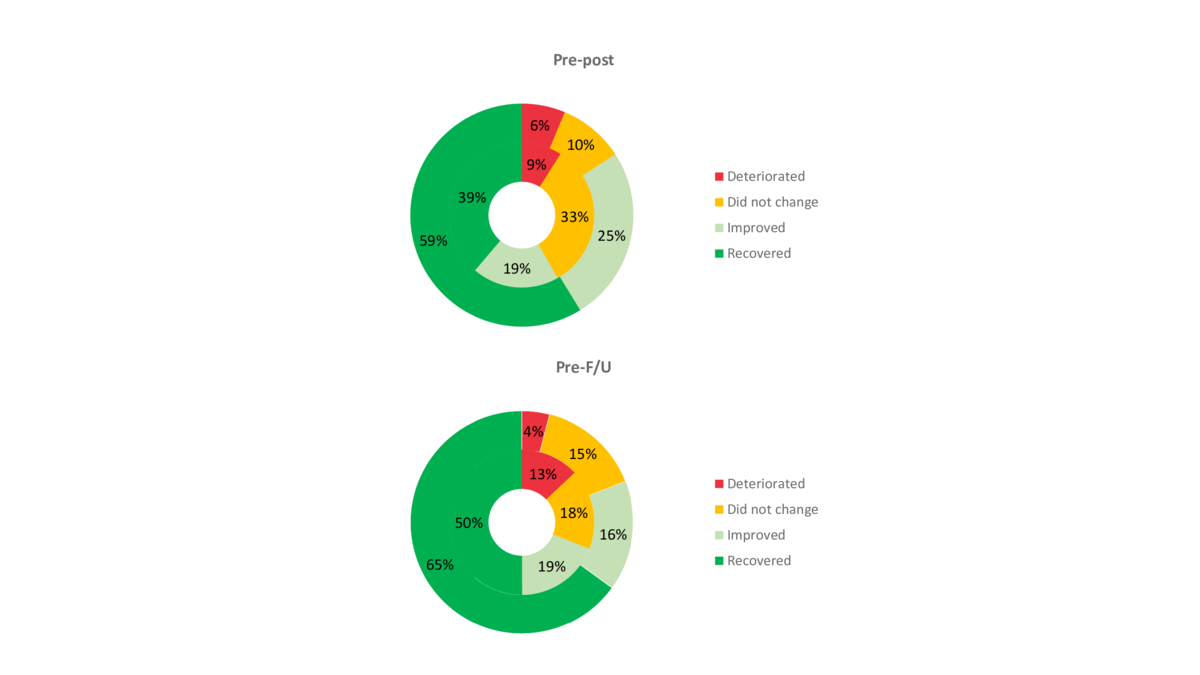
Percentages of participants recovered, improved, did not change, and deteriorated on depression scores (Beck depression inventory-II) in EmotionRegulation (outer circle) and treatment as usual (inner circle). F/U: follow-up.

In the EmotionRegulation group, 84% (85/99) of the patients achieved a functional change in their depression scores, whereas only 58.4% (59/101) did so in the TAU group, and these differences were significant (X^2^_1_=10.5; *P*=.001). In the EmotionRegulation group, 59% (58/99) of participants recovered, 25% (25/99) improved, 10% (10/99) did not change, and 6% (6/99) deteriorated; whereas in the TAU group, 38.6% (39/101) of participants recovered, 18.8% (19/101) improved, 32.7% (33/101) did not change, and 8.9% (9/101) deteriorated (Figure 2). Differences betweeen groups were significant (X^2^_3_=11.7; *P*=.009).

##### Baseline to Follow-Up Changes

In the EmotionRegulation group, 80% (79/99) of the patients achieved a functional change in their depression scores, whereas only 70.3% (71/101) of the patients did so in the TAU group, and these differences were not significant (X^2^_1_=1.6; *P*=.20). In the EmotionRegulation group, 65% (64/99) of participants recovered, 16% (16/99) improved, 16% (16/99) did not change, and 4% (4/99) deteriorated; whereas in the TAU group, 50.5% (51/101) of participants recovered, 19.8% (20/101) improved, 17.8% (18/101) did not change, and 12.9% (13/101) had deteriorated (Figure 2). No significant differences betweeen groups were found (X^2^_3_=3.7; *P*=.30).

#### Changes in Anxiety

##### Baseline to Posttreatment Changes

**Figure 3:**
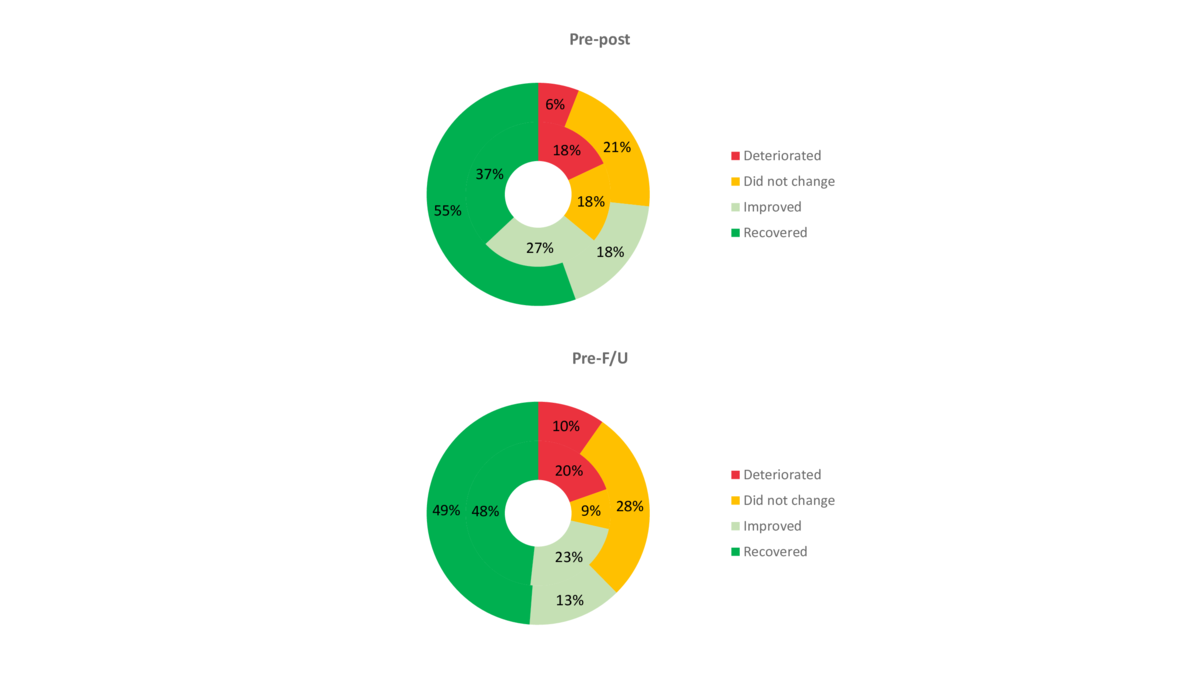
Percentages of participants recovered, improved, did not change, and deteriorated on anxiety scores (Beck anxiety inventory) in EmotionRegulation (outer circle) and treatment as usual (inner circle). F/U: follow-up.

There were no significant differences in the proportion of patients who achieved a functional change in their anxiety scores between the 2 conditions (X^2^_1_=1.2; *P*=.28). However, 73% (72/99) of participants in the EmotionRegulation group achieved a functional change in anxiety and 64.4% (65/101) did so in the TAU group. . In the EmotionRegulation group, 56% (55/99) of participants recovered, 18% (18/99) improved, 21% (21/99) did not change, and 6% (6/99) deteriorated. In contrast, in the TAU group, 36.6% (37/101) of participants were recovered, 26.7% (27/101) were improved, 17.8% (18/101) did not change, and 17.8% (18/101) had deteriorated (Figure 3). Differences betweeen groups were marginally significant (X^2^_3_=7.3; *P*=.06).

##### Baseline to Follow-Up Changes

There were no significant differences in the proportion of patients who achieved a functional change in their anxiety scores between the 2 conditions (X^2^_1_=0.9; *P*=.34). In the EmotionRegulation group, 49% (49/99) of participants recovered, 14% (14/99) improved, 28% (28/99) did not change, and 10% (10/99) deteriorated. In contrast, in the TAU group, 47.5% (48/101) of participants were recovered, 22.8% (23/101) were improved, 8.9% (9/101) did not change, and 19.8% (20/101) had deteriorated (Figure 3). Differences betweeen groups were significant (X^2^_3_=8.2; *P*=.04).

### Treatment Acceptability

Table 5 provides the means and SDs for expectations and opinions about treatment in the completer sample of the EmotionRegulation condition (n=63). As hypothesized, the results indicate that participants reported high scores on all the items measuring treatment expectations (scores between 7.56 and 7.81): logic of the treatment, satisfaction with the treatment, recommending the treatment to other people with similar problems, usefulness of the treatment for other psychological problems, and usefulness of the treatment for one’s specific problem. After receiving the intervention, scores for treatment opinions were generally higher than scores for treatment expectations (scores between 7.67 and 8.24).

A 1-way repeated measures ANOVA showed that, compared with treatment expectations, the opinion of treatment was significantly better for item 1 (logic of the treatment; *F*_1,62_=7.81; *P*=.007; *η^2^* partial=0.11), item 3 (recommending the treatment to other people with similar problems; *F*_1,62_=4.80; *P*=.03; *η^2^* partial=0.07), and item 4 (usefulness of the treatment for other psychological problems; *F*_1,62_=4.92; *P*=.30; *η^2^* partial=0.07). No significant differences were found for item 2 (satisfaction with the treatment; *F*_1,62_=3.05; *P*=.09; *η^2^* partial=0.05) or item 5 (usefulness of the treatment for one’s specific problem; *F*_1,62_=.21; *P*=.65; *η^2^*partial=0.003).

**Table 5:** Means and SDs for expectations and opinions of treatment (n=63).

Item	Expectations, mean (SD)	Opinion, mean (SD)
Treatment is logical	7.65 (1.88)	8.19 (1.62)
Satisfaction with the treatment	7.56 (1.81)	7.90 (1.71)
Recommend to others	7.81 (1.91)	8.24 (1.85)
Usefulness for other psychological problems	7.64 (1.86)	8.05 (1.65)
Usefulness for one’s specific problems	7.76 (1.83)	7.67 (2.13)

## Discussion

### Principal Findings

The objective of this RCT was to explore whether a transdiagnostic internet-delivered protocol (EmotionRegulation) could be effective in treating a wide range of anxiety and depressive disorders, compared with TAU as provided in Spanish public specialized mental health care services. The effectiveness of EmotionRegulation was evaluated on measures of overall anxiety and depression, temperament (ie, BI and BA), and health-related QoL. Finally, expectations and opinions were evaluated to examine the acceptability of EmotionRegulation for the patients. To our knowledge, this is the first RCT to report data on a transdiagnostic internet-delivered protocol for ED in public specialized mental health care.

Regarding our first hypothesis, the ANCOVAs revealed that participants in the intervention group (EmotionRegulation) improved their depression and anxiety symptoms at posttreatment to a greater degree than participants in the TAU group. Regarding the magnitude of the changes at posttreatment, the analyses showed small but significant between-group effect sizes for both depression and anxiety. With regard to measures of temperament, the findings were mixed. Overall, although patients in the EmotionRegulation group showed better scores on the 2 subscales of the BIS and BAS than the TAU group, only the differences on the BAS subscale were significant, favoring the EmotionRegulation group. These results suggest that the intervention tested in this study was able to modify temperament, in line with a mechanistically transdiagnostic approach that assumes the existence of underlying mechanisms that account for the occurrence of specific symptoms. To our knowledge, only 1 study has previously investigated the effect of a mechanistically transdiagnostic treatment on the dimensions of BI and BA [42]. Similar to the results obtained in our study, in the study by Carl et al [42], both BI and BA improved following treatment with the UP compared with a waitlist control group, with small between-group effect sizes found for both the BIS and BAS temperament dimensions. Moreover, these authors showed associations between the gains in temperament dimensions and symptoms of anxiety and depression, particularly for BI, because lower BI scores were associated with greater improvements in anxiety and depressive symptoms. However, a limitation of this study was that the sample size was small, and so the findings were preliminary. Thus, future RCTs of mechanistically transdiagnostic treatments should analyze the extent to which these interventions are able to modify BI and BA and other related temperament dimensions such as negative and positive affect to shed more light on this question [104,105]. Finally, as hypothesized, EmotionRegulation was superior to TAU in improving health-related QoL, with a small but significant between-group effect size observed for the EQ-5D-3L scale. In this regard, it is known that pharmacological treatment could be linked to some variables associated with health-related QoL, such as fatigue and sexual functioning [106]. Therefore, decreases in medication may have indirectly influenced the QoL in some patients. However, these decreases were not measured, and hence, no analyses could be performed to analyze this aspect.

Furthermore, to evaluate the significance of the clinical gains, Jacobson and Truax’s RCI was obtained for the principal outcome measures, that is, BDI-II and BAI.

Consistent with the aforementioned results, significant differences were observed in the proportion of patients who achieved a functional change in depression scores, with a significantly higher number of patients within the functional range in the EmotionRegulation condition (85/99, 84%) than in the TAU group (59/101, 58.4%). Regarding anxiety scores, although no significant differences were observed between the 2 groups in the proportion of patients reaching a functional change, a higher proportion of patients in the EmotionRegulation group (72/99, 73%) than those in the TAU group (65/101, 64.4%) reached a functional change. Furthermore, significant differences were found in the proportion of patients recovered, improved, did not change, and deteriorated, with better general results for the intervention group than for the TAU group. At follow-up, there were no differences in the proportion of patients reaching a functional change in either depression or anxiety. Therefore, it can be concluded that these differences tended to diminish between groups at follow-up.

Regarding the acceptability of EmotionRegulation, expectations about the treatment were high (scores ≥7). After receiving the treatment, compared with expectations, the participants rated the program as significantly more logical, more recommendable for other people with similar problems and more useful for the treatment of other psychological problems. The study of acceptability is important because expectations about treatment have been shown to affect treatment outcomes [107]. Furthermore, because most transdiagnostic internet-delivered protocols have been conducted in community samples [108], it is necessary to continue to explore the acceptability of these interventions in specialized care.

Overall, the findings showed that the transdiagnostic internet-delivered protocol tested in this RCT was more effective than TAU for the treatment of anxiety and depressive disorders in public specialized mental health care. On the one hand, the results show that EmotionRegulation led to greater improvements at posttreatment, and these gains were maintained at follow-up. Regarding TAU, the results reveal that patients undergoing TAU also experienced improvement over time, but it was slower and less pronounced than in patients receiving EmotionRegulation. The generally lower intensity of TAU (ie, lower frequency of therapy sessions) and the fact that for most patients TAU was limited to pharmacotherapy (with no access to psychological treatment) might partly account for these results. On the other hand, as anticipated, and in line with previous studies conducted by our research group using the same treatment platform [94,109], scores on expectations and opinions demonstrated EmotionRegulation’s acceptability for participants.

The results obtained in this RCT have implications for both research and clinical practice, especially in the context of public specialized mental health care. First, the findings obtained in this study support the effectiveness of a mechanistically transdiagnostic internet-delivered protocol for the treatment of EDs, and they contribute to the literature in this particular field [23,27,36,110-112]. Specifically, a combination of the components of the UP and DBT regulation skills was found to be more effective than TAU in treating ED. Second, as far as we know, this is the first study to explore a transdiagnostic internet-delivered treatment in public specialized mental health care. As mentioned earlier, most research on transdiagnostic web-based treatments has been conducted in community settings, with a few of these studies carried out in primary care [108]. The results showed that the intervention was found to be more effective than TAU on the measures of generic depression, anxiety, and QoL. These results are consistent with the literature showing the superiority of CBT over TAU. For instance, a meta-analysis showed that CBT outweighed TAU, with effect sizes in the medium range on measures of generic anxiety (Hedges *g*=0.70) and depression (Hedges *g*=0.69) [70]. As surprising as these data might seem, the truth is that current public mental health services still have to deal with a number of barriers that hinder appropriate care delivery, such as excessive waiting times to access mental health care [60], low frequency of sessions [113], or inadequate follow-up care [56]. Moreover, the lack of training in evidence-based treatments among professionals further adds to this problem [114]. Finally, in Spain, several RCTs have been conducted using the internet to provide evidence-based treatments, showing that they are effective for the treatment of ED and, in particular, depression, in community samples [94] and primary care [115,116], and others are underway [117]. This study demonstrated that an internet-delivered protocol for ED was effective in public specialized care, a setting with a high demand, but much less explored, thus adding to the literature on these treatments for ED. Furthermore, the use of TAU as the control condition may help to answer the question of “whether a new treatment or an evidence-based psychotherapy really surpasses in outcome effects what is ordinarily done at a given clinic” [118], thus helping to make clinicians, researchers, and policy makers aware of the limitations and aspects that should be improved in this specific setting.

### Limitations

Although the results of this RCT are promising, they should be interpreted in light of the following limitations. First, although several measures were taken to minimize attrition (eg, guidance was provided to all the patients participating in EmotionRegulation), the number of patients who dropped out of the study was high (around 35%). However, this proportion was close to what is typically observed in the literature on internet-delivered psychological treatments (ie, approximately 30%-35%) [71]. Moreover, attrition in the TAU condition was similar (34/108, 31.5%). Second, this RCT was not powered to detect differences in disorder-specific measures (ie, for GAD, PD or AG, SAD, and OCD). Therefore, future studies that meet the minimum levels of statistical power to detect differences in these measures are warranted. Third, although the frequency of the session in TAU was low and most patients in this condition were receiving only pharmacotherapy (as observed by the researchers), these data were not monitored during the trial. Finally, the results for acceptability (ie, expectations and opinions) might not be entirely representative because data from patients who dropped out of the intervention were not included in these analyses.

### Conclusions and Future Directions

The effectiveness of a mechanistically transdiagnostic internet-delivered protocol for ED was compared with TAU in public specialized mental health care. Although the results are promising, more research in specialized care should be conducted to extend the findings obtained in this study. First, research on predictors and moderators of treatment outcomes and dropout in this specific setting can help to delineate the profiles of participants who are more likely to benefit from these treatments and help to answer the classic question, “what treatment, by whom, is most effective for this individual with that specific problem, under which set of circumstances?” [119]. Moreover, we believe that the integration of principles from different evidence-based protocols (eg, components of the UP and DBT) can be a powerful strategy that should guide future research on evidence-based psychotherapy. This strategy is in line with process-based CBT and the new foundational question proposed by Hofmann and Hayes [49] ( ie, “What core biopsychosocial processes should be targeted with this client given this goal in this situation, and how can they most efficiently and effectively be changed?”) . Second, despite the huge advances experienced by the field of internet-delivered treatments in the past two decades, high dropout rates remain a major challenge in the field. To continue to improve current and future internet-delivered interventions, future studies should strive to include dropouts in the analysis of acceptability using both qualitative and quantitative approaches. Moreover, to ensure the integrity, quality, and replicability of these studies, adherence to existing research guidelines is of paramount importance in this endeavor. Third, although we did not assess the acceptability of clinicians involved in the RCT (ie, psychiatrists, clinical psychologists, and nurses), it is worth mentioning that some of them refused to participate in the recruitment process, which might reflect negative attitudes toward internet-delivered interventions among these professionals. In this scenario, research efforts should be made to inform clinicians and state holders about the benefits of internet-delivered treatments, especially because they are seen as authority figures and, therefore, their attitudes can have a major impact on patients’ perceptions. Finally, although the need for efficacy studies is out of doubt, we believe that it is of paramount importance to conduct more implementation research [120]. These studies may provide a much deeper understanding of implementation variables that can either facilitate or hamper the effective uptake of evidence-based protocols in real clinical practice, such as the attitudes of clinicians and other professionals toward internet-delivered treatments, or economical and logistic aspects that are difficult to implement.

## Appendix

Multimedia Appendix 1 Descriptive statistics and effect sizes for disorder-specific measures.

Multimedia Appendix 2 CONSORT-eHEALTH (V 1.6.1).

## Abbreviations

AG: agoraphobia
ANCOVA: analysis of covariance
ANOVA: analysis of variance
BA: behavioral activation
BAI: Beck Anxiety Inventory
BAS: behavioral activation scale
BDI-II: Beck depression inventory, 2nd edition
BI: behavioral inhibition
BIS: behavioral inhibition scale
CBT: cognitive behavioral therapy
CONSORT: Consolidated Standards of Reporting Trials
DBT: dialectical behavioral therapy
DD: dysthymic disorder
DSM-IV: Diagnostic and Statistical Manual of Mental Disorders, Fourth Edition
ED: emotional disorder
EQ-5D-3L: EuroQoL-5D-3L questionnaire
GAD: generalized anxiety disorder
ITT: intention-to-treat
MCAR: missing completely at random
MDD: major depressive disorder
MINI: mini-international neuropsychiatric interview
ML: maximum likelihood
NOS: not otherwise specified
OCD: obsessive compulsive disorder
PD: panic disorder
QoL: quality of life
RCI: reliable change index
RCT: randomized controlled trial
SAD: social anxiety disorder
SI: societal index
TAU: treatment as usual
UP: Unified Protocol
VAS: visual analog scale
