# Supplementary material for: Effectiveness of a Transdiagnostic Guided Internet-Delivered Protocol for Emotional Disorders Versus Treatment as Usual in Specialized Care: Randomized Controlled Trial
Source: J Med Internet Res. 2020 Jul 7;22(7):e18220. doi: 10.2196/18220 (PMC7381075; doi:10.2196/18220)
Supplement: Multimedia Appendix 1 [file jmir_v22i7e18220_app1.docx]

|  | EmotionRegulation | | | TAU | | |
| --- | --- | --- | --- | --- | --- | --- |
|  | Pre-T, *M* (*SD*) | Post-T, *M* (*SD*) | F/U, *M* (*SD*) | Pre-T, *M* (*SD*) | Post-T, *M* (*SD*) | F/U, *M* (*SD*) |
|  |  |  |  |  |  |  |
| PSWQ (n = 50) | 68.33 (6.19) | 62.99 (7.97) | 61.31 (9.88) | 67.58 (7.43) | 61.87 (7.00) | 60.31 (8.07) |
| PDSS-SR (n = 43) | 12.17 (5.08) | 7.73 (5.31) | 8.14 (4.29) | 11.89 (6.36) | 7.42 (4.77) | 9.88 (6.45) |
| SIAS (n = 8) | 50.75 (9.03) | 45.79 (9.93) | 38.04 (7.74) | 51.00 (10.42) | 50.10 (6.13) | 44.20 (6.23) |
| OCI-R (n = 20) | 19.25 (9.47) | 12.02 (10.80) | 12.87 (7.32) | 34.75 (18.97) | 29.78 (20.84) | 20.84 (13.21) |

Table 1. Means and standard deviations for disorder-specific measures

PSWQ: Penn State Worry Questionnaire; PDSS-SR: Panic Disorder Severity Scale – Self-reported; SIAS: Social Interaction Anxiety Scale; OCI-R: Obsessive-Compulsive Inventory – Revised

Table 2. Within-group effect sizes for disorder-specific measures

|  | EmotionRegulation | | TAU | |
| --- | --- | --- | --- | --- |
|  | Pre-post,  *d* (95% CI) | Pre-F/U, *d* (95% CI) | Pre-post,  *d* (95% CI) | Pre-F/U, *d* (95% CI) |
|  |  |  |  |  |
| PSWQ (n = 50) | .83 (.41 to 1.26) | 1.10 (.57 to 1.62) | .75 (.34 to 1.15) | .95 (.47 to 1.43) |
| PDSS-SR (n = 43) | .85 (.44 to 1.25) | .77 (.30 to 1.23) | .67 (.27 to 1.08) | .30 (-.17 to .77) |
| SIAS (n = 8) | .40 (-2.14 to 2.93) | 1,02 (-.95 to 2.99) | .06 (-1.19 to 1.31) | .47 (-.95 to 1.90) |
| OCI-R (n = 20) | .68 (.18 to 1.18) | .60 (-.22 to 1.42) | .24 (-.03 to .52) | .68 (.02 to 1.35) |

PSWQ: Penn State Worry Questionnaire; PDSS-SR: Panic Disorder Severity Scale – Self-reported; SIAS: Social Interaction Anxiety Scale; OCI-R: Obsessive-Compulsive Inventory – Revised
